# The pedagogical rehabilitation in the construction of the alphabetic writing of children with cochlear implant

**DOI:** 10.1016/j.bjorl.2024.101457

**Published:** 2024-06-08

**Authors:** Rogério Hamerschmidt

**Affiliations:** a Universidade Federal do Paraná, Departamento de Otorrinolaringologia, Curitiba, PR, Brazil; b Universidade Federal do Paraná, Programa de Pós-Graduação em Clínica Cirúrgica, Curitiba, PR, Brazil

Children's cognitive development and socialization are related to language acquisition.^1^, ^2^ Writing is not a natural process inherent in human development, but a taught process with deep cultural roots. The act of reading and writing is not limited to learning to decode and encode graphemes, recognizing letters and words, but to produce meaning about the text and understand writing in its various functions.^3^, ^4^

Pedagogical rehabilitation (PH) in hearing health does not aim at teaching school content, but at the child's thinking and creativity, challenging them to use writing in a functional way, even before learning to read and write (literacy process). It enhances the production of meanings in their social interaction and stimulates cognitive functions, especially auditory memory and language, fundamental elements to work on the prerequisites of writing.^5^

The child with cochlear implant (CI) should also receive motor and sensory stimulation in the pedagogical care to help develop skills for the act of writing (manual dexterity/visual motor skill) and cognitive skills, as it involves higher psychological functions, attributes necessary to produce meaningful writing.

PH in auditory rehabilitation offers significant learning conditions for the child with cochlear implant when experienced through children's games, stories, songs, nursery rhymes and games. Due to the richness of natural stimulation and spontaneity that playfulness provides to child development. It helps children to internalize new perceptions and cultural forms of their external environment, whose input will produce determinant meanings for the intelligible use of speech and, consequently, of writing.

It is important that hearing rehabilitation centers also offer children quality PH together with speech therapy and psychological care, expanding the service also to the demands of education and family professionals because they are directly contextualized to the child's daily life and are rich collections of natural stimuli, enhancing their school performance.

## Conflicts of interest

The author declares no conflicts of interest.
